# Editorial: Advances in conservation and utilization of plant genetic resources

**DOI:** 10.3389/fpls.2024.1468904

**Published:** 2024-08-07

**Authors:** Svein Øivind Solberg, Maarten van Zonneveld, Axel Diederichsen

**Affiliations:** ^1^ Department of Agriculture, Inland Norway University of Applied Sciences, Elverum, Norway; ^2^ World Vegetable Center, Shanhua, Taiwan; ^3^ Plant Gene Resources of Canada, Agriculture and Agri-Food Canada (AAFC), Saskatoon, SK, Canada

**Keywords:** conservation, crop wild relatives, diversity, evaluation, food security, genebank, plant genetic resources, seed bank

## Background

We made a broad call for perspective papers, systematic reviews, meta-analyses, or traditional research papers on topics related to conservation and use of plant genetic resources. We invited to present advances in characterization and evaluation, strategies to improve gene bank operations and collaboration, new tools for managing and sharing information, or novel knowledge of conservation gaps. We especially encouraged contributions on underutilized crops and crops wild relatives. We received 58 positive responses, whereof 20 finalized their submissions and passed the review process with a total of 125 authors included in the accepted papers. Our motivation was to assemble contributions underlining that genetic resources are essential for crop improvement, which is achieved via plant breeding and release of new varieties that farmers cand access and use. We see that future crops need to produce high and stable yields but also be of high nutritional quality. They must adapt to shifting climates and support a sustainable agricultural or horticultural production avoiding negative environmental impacts. In addition, we need to contribute to efforts to avoid the loss of biodiversity, including the genetic resources of crop plants and crop wild relatives, which are in our focus. We are aware that there is a large body of research and literature on this, because plant breeders in the late 19^th^ century already imitated research and conservation activities which serve the same purpose and they recognized the loss of biodiversity, albeit not using this term. However, the global biodiversity crisis has become much bigger and globally recognized and any additional step, any additional insight addressing this topic is worth to be shared. At the same time more and more genomic tools and other technologies are available to add to our understanding of diversity and can support conservation and target promising germplasm for further research and breeding. We hope this special edition will be a small additional step in a positive direction, part of the evolutionary process of coping with the grand challenges we all face.

## Conservation

Six papers focused on conservation, which included conservation methods and priorities as well as core collections. Of these, one is a review paper and five are research articles.

Subramanian et al.’s review on *Brassica* biodiversity conservation discusses issues related to distribution of accessions, conservation methods, technical hurdles and future avenues for research. Despite that there are more than 80,000 *Brassica* accessions conserved across 81 countries the authors see that the diversity of the conserved taxa is limited in most countries, which they see may lead to biodiversity loss in the longer run. In addition, they see practical challenges as taxonomic issues in the conservation system.

The research paper from Weise et al. focuses on the rapeseed (*Brassica napus* L.) gene pool that is conserved in European genebanks. They highlight that most of these species are underrepresented in current collections and that many of the natural distribution areas are not covered as it stands now. By using niche modelling they further illustrate how climate change may affect the species’ distribution ranges. The authors suggest to further develop the conservation strategies for the rapeseed gene pool and propose a list of priority species that should be targeted for collecting missions.

Carvajal-Yepes et al. made a work to identify genetically redundant accessions in the world’s largest cassava (*Manihot esculenta*) collection with 5,302 accessions maintained at the International Center for Tropical Agriculture (CIAT). Am empirical distance threshold methodology was applied with two types of molecular markers (SNP and SilicoDArT). The results showed 2,776 (SNP) and 2,785 (SilicoDArT) accessions were part of accession clusters. By comparing passport and historical characterization data clusters of genetically redundant accessions the authors provided a roadmap for genebank curators to assess redundancy within collections and/or identifying subsets of genetically distinct accessions.

Almeida et al. presented a methodology for landrace threat assessment which can assist in setting priorities for conservation. The methodology of this work was in line with the IUCN Red Listing judgement for wild species and involves the collation of time series information on population range and trends. Unlike for wild species the information used here involved farmers and market actors. The authors conclude that the archived information can be compared to a standardized set of threat criteria with a set threshold level and the methodology can be applied to any crop and geographical scale.

Dos Santos et al. developed a core collection of the cassava (*Manihot esculenta* Crantz) germplasm bank in Brazil based on morphological traits, agronomic traits, and genotypic data from 20k single-nucleotide polymorphisms (SNPs). Out of the 1,486 accessions in the germplasm bank a consolidated core with 204 accessions was suggested, which is 14% of the complete collection. This core collection showed less genetic variation but retained over 97% of the allelic richness compared to the complete collection. The authors see that the core approach provides a robust and representative resource for further research and breeding in cassava.

Bernad et al. examined the genetic diversity and population structure of the newly introduced two-row spring European Heritage Barley collection. This is a small collection which consists of 363 spring-barley accessions mainly from Northern Europe and include both landraces (~14%), old cultivars (~18%), elite cultivars (~67%) and accessions with unknown breeding history (~1%). The authors used 26,585 informative SNPs based on 50k iSelect array data and the results showed that the collection could be subdivided into three main clusters. The clusters were primarily based on the accession’s year of release. Furthermore, power analysis identified a core with 230 genotypically and phenotypically diverse accessions, which shows that the collection represents a high diversity and can be a resource for research and breeding.

## Characterization and evaluation

Twelve research articles from recent characterization and evaluation projects are presented, which included a range of crops, methods and aims and showing how molecular markers and genomic tools can help to identify promising germplasm for further phenotyping and evaluation.

Gomez-Galvez et al. examined more than 500 olive (*Olea europaea* L.) genotypes from different regions in Spain based on 96 EST-SNP markers and identified 173 new genotypes. Based on this work the number of distinct Spanish genotypes documented in the World Olive Germplasm Bank of IFAPA, Córdoba increased from 269 to 427 accessions. In addition, a new diversity hot spot was identified in the northern regions of La Rioja and Aragon. According to the authors this adds to the great diversity already described in Spanish olive germplasm. The authors highlight the risk of genetic erosion given the expansion of modern olive cultivation with only a few cultivars and argue that conserving a broad range of genotypes will be crucial to meet the future challenges of olive cultivation. To further enhance the conservation and use of these accessions, the World Olive Germplasm Bank of Córdoba was recognized in June 2024 to become an international collection following Article 15 of the International Treaty on Plant Genetic Resources for Food and Agriculture.

Adhikari et al. conducted a genomic characterization using more than 45k SNPs of the 1,041 *Aegilops* accessions preserved in the Wheat Genetics Resource Center (WGRC) collection. The *Aegilops* genus contains a range of crop wild relative species which are regarded as critical for future wheat crop improvements. The authors present phylogenetic tree and principal component analyses that showed some species overlap but also pathways of species evolution and diversification. The high genetic diversity identified among the species indicate their importance as genetic resources for future wheat breeding. For genebank curation the study found 49 misclassified and 28 sets of redundant accessions in the WGRC collection.

Berkner et al. worked on genomic prediction to identify winter wheat accessions that can be used to breed varieties with a combined high protein and high lysine content. Their point of departure was a large-scale screening that was conducted back in the 1970s at the Leibniz Institute of Plant Genetics and Crop Plant Research in Germany. Additionally, they used a genomic dataset generated in 2022. The datasets were curated, four genomic prediction approaches were compared, and the best model was used to predict the traits of interests. Out of the 7,651 accessions included in the predictions, five accessions were highlighted as combining outstanding high protein content with high lysine content.

Chao et al. examined the genetic diversity and population structure of annual medicks (*Medicago* spp.) from the Crimean Peninsula of Ukraine collected in 2008. The colleting mission was done to fill gaps at National Plant Germplasm System in USA and 102 accessions from 10 species were collected. The authors present the results from characterization work, which included 24 phenotypic descriptors and a 3k SNP marker set developed for lucerne (alfalfa). The results showed a high reproducibility between single and pooled biological replicate leaf samples, which indicates that sampling individual plants for these mostly self-pollinating species is sufficient. According to the authors the phenotypic descriptors and the applied SNP marker set was useful in assessing the population structure.

Liu et al. conducted a complete mitochondrial genome characterization and phylogenetic analysis of the endangered species *Prunus pedunculata* in China. The authors highlight that the results provide a basis for understanding the evolution of the genetic background and genetic breeding of Prunus.

Abondano et al. compared single plants, multiple plants, and DNA pools sampling strategies for DArTseq genotyping common bean (*Phaseolus vulgaris* L.) landraces from the Alliance Biodiversity and CIAT gene bank. They concluded that pooling tissue from 25 individual plants per accession was a viable approach for characterizing germplasm compared to genotyping individual plants separately by balancing genotyping effort and costs. The results add valuable insights for characterization of collections and in marker-trait association studies.

Loarca et al. evaluated shoot-growth variation in a diversity panel of 695 accessions of carrot (*Daucus carota* L.) from the United States Department of Agriculture National Plant Germplasm System. They found phenotypic variability for seedling emergence and early-season canopy coverage, which is indicating quantitative inheritance and potential for improvement through plant breeding. Accessions with high emergence and vigorous canopy growth are of immediate use to breeders targeting stand establishment, weed-tolerance, or weed-suppressant carrots, which is of advantage to the organic carrot production. In a second paper Loarca et al. evaluated flowering habit trait of the same accessions. They found a high broad-sense heritability for biennial flowering habit which indicates a strong genetic component of this trait.

Li et al. conducted phylogeographic analysis of the native grass *Elymus nutans* using microsatellite markers and covering 361 individual plants across 35 populations from the Qinghai-Tibetan plateau. The species has pastoral and environmental importance, and the study unveiled a notable degree of genetic diversity. Correlations were established between external environmental factors and effective alleles potentially linked to glutathione S-transferases T1 or hypothetical proteins, which are affecting environmental adaptation.

An et al. analyzed the genetic diversity and structure of a perennial evergreen tree *Albizia odoratissima* using 16 simple sequence repeat markers and covering 280 individuals across 10 populations from Hainan Island and mainland China. The genetic diversity of Hainan population was lower than that of the mainland population Furthermore there were significant differences in the genetic structure between Hainan and mainland populations.

Lu et al. examined the diversity of an herbaceous climber *Dioscorea bulbifera* native to Africa and Asia and locally used as vegetable and medicine. The study included accessions from mainland China and Taiwan that were analyzed using SSR marker and phylogenetic analyses. They showed structural features across accessions and three distinct clades indicating potential genetic divergence among populations from different geographic regions in China and Taiwan.

Yang et al. present a characterization work on an unusual type of horny goat weed (*Epimedium koreanum* Nakai) discovered in the Jilin Province in China. Horny goat weed is a well-known traditional Chinese medicinal herb that is collected from natural habitats. The newly discovered type had much higher number of leaflets than commonly found (27 compared to 9). By DNA barcoding this novel type was identified as *E. koreanum.* Parallel RNA-seq analysis showed 1171 differentially expressed genes compared to wild type. Due to a decreasing natural population cultivation could be an alternative source for utilization and this high leaf-yielding *Epimedium* plant could be potentially used in breeding or cultivation.

## Breeding and seed systems

Two research articles are presented, which includes one article on international breeding collaboration to achieve frost tolerance in potato, and one article overviewing breeding seeds as part of the official seed system in India.

Arcos-Pineda et al. report the results from an international breeding project using a wild potato relative *Solanum commersonii* that resulted in two new frost-tolerant native potato cultivars for the Andes and the Altiplano. The project was a collaboration between partners from USA and Peru as well as the International Potato Center (CIP). After 8 years of breeding the two new cultivars were released. The project shows that international collaboration and the use of valuable genetic diversity can produce results of importance for food security.

Chand et al. provide a retrospective overview on the seed production system of lucerne (*Medicago sativa* L.) in India. Out of 14 lucerne varieties released and notified over the past 24 years, only nine entered the seed chain. The varietal replacement rate was found to be moderate, and the authors present a holistic overview and a way forward to develop more varieties and improved production of certified seeds in the country.

## Summary

The provided contributions cover a good mix of topics related to conservation and utilization of plant genetic resources from across the globe, but we miss reports from Africa. We know that more and more research is being done on neglected and underutilized species in this region. On the other hand, we note active work on these matters especially in China. We further note a strong molecular emphasis but that many challenges of managing genetic resources be in genebanks or in *in situ* situations are the same as they were before.

## Author contributions

SS: Writing – original draft, Writing – review & editing. MZ: Writing – original draft, Writing – review & editing. AD: Writing – original draft, Writing – review & editing.

